# Markets, voucher subsidies and free nets combine to achieve high bed net coverage in rural Tanzania

**DOI:** 10.1186/1475-2875-7-98

**Published:** 2008-06-02

**Authors:** Rashid A Khatib, Gerry F Killeen, Salim MK Abdulla, Elizeus Kahigwa, Peter D McElroy, Rene PM Gerrets, Hassan Mshinda, Alex Mwita, S Patrick Kachur

**Affiliations:** 1 Ifakara Health Research and Development Centre, P O Box 78373, Dar es salaam, Tanzania; 2 Centers for Disease Control and Prevention, Division of Parasitic Diseases National Center for Zoonotic, Vector-Borne & Enteric Diseases Coordinating Center for Infectious Diseases, Strategic and Applied Sciences Unit, Malaria Branch, 4770 Buford Highway, NE Mailstop, F-22 Atlanta, Georgia 30341, USA; 3 Durham University, Institute of Ecosystems Science, School of Biological and Biomedical Sciences, South Road, Durham, DH1 3LE, UK; 4 Centers for Disease Control and Prevention, President's Malaria Initiative, American Embassy, P O Box 9123, Dar es salaam, Tanzania; 5 Max Planck Institute for Social Anthropology, PO Box 11 03 51, 06017 Halle/Saale, Germany; 6 Ministry of Health and Social Welfare, National Malaria Control Programme, P O Box 38112, Dar es salaam, Tanzania

## Abstract

**Background:**

Tanzania has a well-developed network of commercial ITN retailers. In 2004, the government introduced a voucher subsidy for pregnant women and, in mid 2005, helped distribute free nets to under-fives in small number of districts, including Rufiji on the southern coast, during a child health campaign. Contributions of these multiple insecticide-treated net delivery strategies existing at the same time and place to coverage in a poor rural community were assessed.

**Methods:**

Cross-sectional household survey in 6,331 members of randomly selected 1,752 households of 31 rural villages of Demographic Surveillance System in Rufiji district, Southern Tanzania was conducted in 2006. A questionnaire was administered to every consenting respondent about net use, treatment status and delivery mechanism.

**Findings:**

Net use was 62.7% overall, 87.2% amongst infants (0 to1 year), 81.8% amongst young children (>1 to 5 years), 54.5% amongst older children (6 to 15 years) and 59.6% amongst adults (>15 years). 30.2% of all nets had been treated six months prior to interview. The biggest source of nets used by infants was purchase from the private sector with a voucher subsidy (41.8%). Half of nets used by young children (50.0%) and over a third of those used by older children (37.2%) were obtained free of charge through the vaccination campaign. The largest source of nets amongst the population overall was commercial purchase (45.1% use) and was the primary means for protecting adults (60.2% use). All delivery mechanisms, especially sale of nets at full market price, under-served the poorest but no difference in equity was observed between voucher-subsidized and freely distributed nets.

**Conclusion:**

All three delivery strategies enabled a poor rural community to achieve net coverage high enough to yield both personal and community level protection for the entire population. Each of them reached their relevant target group and free nets only temporarily suppressed the net market, illustrating that in this setting that these are complementary rather than mutually exclusive approaches.

## Background

It is estimated that malaria is responsible for 515 million clinical attacks worldwide, 70% of these events are concentrated in Africa [1]. Young African children and pregnant women bear brunt of the burden and at least 18% of childhood mortality on the continent is due to malaria [2]. More encouragingly, the fact that insecticide-treated nets (ITN) prevent malaria has been irrefutably documented [3,4]. The Roll Back Malaria Partnership and Millennium Development Goals (MDG), therefore, aim to achieve 80% ITN use amongst pregnant women and children below five years of age in Africa, while the US President's Malaria Initiative (PMI) is even more ambitious, aiming for 85% use amongst these same population categories [5-7]. However, there is growing consensus that this important intervention will only achieve its full potential to prevent malaria if at least one third of the entire population sleeps under ITN, as well as the vast majority of the most vulnerable groups such as pregnant women and young children [8-12]. This is because residents are protected by not only personal use of ITNs but also by the community-wide effect that their neighbours nets have on mosquito populations. Much as there is increasing call for rapid and sustained achievement of high ITN coverage targeting entire populations [9-11,13], including non-vulnerable adults and older children, delivery mechanisms by which this noble goal can be achieved are still actively debated [14-16]. Until recently, public debate has largely focussed upon the comparative merits of free and market-based strategies for deploying ITNs [15-18]. While spirited debate over such a potentially important public health issue is welcome [15], it carries a risk that policy makers and donors will perceive a false dichotomy between free and market-based strategies for promoting ITNs. If so, they may overlook an important opportunity to implement complementary strategies for rapidly increasing and maintaining high levels of ITN ownership and use.

Tanzania has been a front-line country for testing the efficacy [19] and effectiveness [20] of ITNs, and has developed a nationwide implementation strategy based on in-country experience [21]. Notably, it was also the first country in which a large-scale cost-sharing scheme for distributing subsidized ITNs was evaluated and shown to improve child survival under programmatic conditions [20]. When Tanzania first decided to take ITNs to scale, mosquito nets were almost exclusively supplied through commercial retailers bundled with insecticide-treatment kits subsidized by the public sector [21]. In 2004, the National Malaria Control Programme (NMCP) introduced a voucher subsidy for pregnant women as part of a nation-wide programme to prevent malaria by enhancing coverage of pregnant women and the young children who share their sleeping spaces during and after the pregnancy. In addition to this national programme, NMCP also assisted a small number of districts including Rufiji on the south-central coast to distribute free bundled nets to under-fives through a child health campaign in mid-2005 with support from partner organizations including UNICEF and the Tanzanian Red Cross [22].

The Interdisciplinary Monitoring Project for Anti-malarial Combination Therapy (IMPACT) had been implementing and evaluating effects on drug resistance of sulphadoxine-pyrimethamine (SP) combined with artesunate (SP+Art) for routine treatment of malaria in Rufiji district southern Tanzania between 2000 – 2006 [23]. Annual household surveys, which included net ownership, use and source, were conducted as a routine part of this study. The coincidence of the unsubsidized market, voucher subsidies and free distribution happening at the same time and place created an opportunity to evaluate the interactions between these major and apparently inconsistent ITN delivery strategies – the primary focus of the ongoing debates. This paper presents results from this assessment and show that these combined strategies complemented rather than competed with each other.

## Methods

### Study area and population

**Figure 1:**
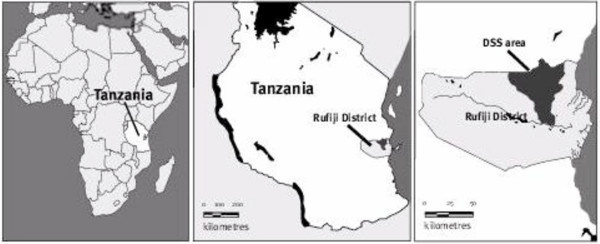
Location of the study area.

Rufiji district lies in southern Tanzania about 178 km south of Dar es Salaam, the country's primary commercial centre and biggest city (Figure 1). The Demographic Surveillance System (DSS) site in which this survey was conducted is composed of 31 villages with an area of 1,813 km^2 ^and population of about 85,000 people [24]. It is low-lying (<500 m above sea level) and most of its surface area lies within the fertile flood plain of Rufiji river. Rufiji typically experiences a long rainy season between February and May and a shorter, less intense one from October to December. The majority of the population in this area belongs to Ndengereko tribe. Other important ethnic groups include the Matumbi, Nyagatwa, Ngindo, Pogoro and Makonde. Islam is the predominant religion in the community and commonest language spoken in the area is Kiswahili, consistent with the rest of the country. The main economic activity is subsistence farming of crops such as rice, cassava, oranges, mangoes, cashews, papayas and coconuts. Farms are often located some distance from the family home and rely on periodically flooded alluvial soils. Residents often stay in seasonal makeshift dwellings at farms, especially during rice growing season of February to July. A significant number of people are also engaged in artisanal fishing, charcoal burning, logging, carpentry and small scale trading. All study villages are located on the northern side of Rufiji River along Dar-es-salaam – Kilwa highway (Figure 1). Most of these villages are quite isolated in the interior of the district and are connected to the highway by unpaved roads that are often impassable during long rains. Most houses have wood-framed mud walls with thatched or corrugated roofs. Common water supplies are communal boreholes, natural spring or river water, and hand-dug wells.

Malaria is among the biggest health problem in the area reported by health system and perceived by local community [25]. It is caused largely by *Plasmodium falciparum*, primarily transmitted by *Anopheles gambiae*, *Anopheles arabiensis *and *Anopheles funestus *vector mosquitoes. Transmission in this area is categorized as intense and perennial [24]. Prompt recognition and timely treatment with sulphadoxine-pyrimethamine (SP) combined with artesunate and the distribution of ITNs to young children and pregnant women in particular were the two priority malaria control measures in the district at the time.

Nets have been available in most retail shops existing in these villages for more than a decade, typically bundled with insecticide. Residents use these retailers to buy their nets and insecticide as they do for other household goods. The normal retail price for a 6 foot × 6 foot net at the time of the study was 6,000 Tanzanian shillings (equivalent to US$ 4.65) and a sachet of insecticide sold at TShs 500 (equivalent to 40 US cents). A voucher subsidy for nets to be used by pregnant women and their newly born babies was introduced under the Tanzania National Voucher Scheme (TNVS) in the study area at the end of 2004. The value of the voucher was fixed at TShs 3,250 (equivalent to US$ 2.5) and vouchers were issued to pregnant mothers attending clinic by Reproductive and Child Health staff. The voucher recipient was then entitled to purchase an insecticide treated net at reduced cost by presenting the voucher and paying the price difference to the contracted retailers. Distribution of nets free of charge to under-five children was implemented through the national child vaccination campaign in July 2005 lasting for three days. Every child below five years presenting for vaccination against measles, treatment of helminths and vitamin A supplementation received a free bed net bundled with an insecticide treatment sachet. Additionally, a small number of interviewees received nets at no cost from a variety of sundry sources, including small-scale donations, relatives and friends.

### Study design and data collection

A survey on which this paper is based was conducted between June and August 2006. A total of 2,000 households were randomly selected from DSS Household Registration Books (HRB), of which 1,752 were completed with 6,331 respondents. In each visited household, every registered and consenting member who was available on the day of the visit was interviewed using a structured questionnaire written in Kiswahili. The questions were pre-tested in 30 households before being finalized and deployed. Data collectors had been used before for similar activities conducted by the project in 2001, 2002, 2004 and 2005, but were nevertheless retrained for three days for this particular survey. Questions written in the questionnaires included date of interview, sex of the respondent, net ownership, whether respondent had slept under net the night preceding the interview, how was the net obtained, whether the net had been treated before, and when was it last treated together with characteristics of houses and households including asset ownership. Dates of birth of household members were already available in the DSS data base. Each completed questionnaire was inspected by the study supervisor who selected a sample of forms from each week's for authentication in the field. Ethical approval for the study was obtained from Centres for Disease Control and Prevention (CDC) of the USA and the Ifakara Health Research and Development Centre, the Medical Research Coordination Committee of the National Institute for Medical Research and Tanzania Commission on Science and Technology of Tanzania.

### Data management and analysis

All completed forms were sent to a central data processing unit and double entered using Microsoft (Redmond, WA) FoxPro^® ^software. Data managers developed automated routines to identify discrepancies and executed some simple consistency and range checks which were resolved by referring to original data forms. All data were cleaned and analysed using STATA Version 9.0 (STATA Corporation). This programme was used in computing frequencies, in doing Chi square tests and calculating 95% Confidence Intervals for examining the existence of real differences in net use, sources used to get the nets and treatment of nets for different population groups described in this paper. A wealth index was constructed using principal component analysis for each household based on members owned assets and housing characteristics as described in detail elsewhere [23]. Concentration curves were plotted and concentration indices calculated by assigning these asset index scores to their respective households' members [26-28] using Microsoft Excel software.

## Results

Net use varied across population age groups (Pearson χ^2 ^(d.f. = 12) = 839.9253; P < 0.001) with excellent targeting of high coverage to the most vulnerable groups (Table 1). Infants (0–12 months) had the highest proportion of net use in the study area, with >85% using any net the previous night, exceeding the targets of the Millennium Development Goals (MDG), Roll Back Malaria (RBM) and the US President's Malaria Initiative (PMI) for ITN use [5-7]. It is true that these targets have only been exceeded in terms of any net and that approximately half of these were not recently treated. However, long-lasting insecticide formulations [29,30] for factory pre-treatment or bundling with all nets made in Tanzania were introduced as of March 2007 so these documented levels of coverage with any net should practically translate such achievements into *de facto *ITN coverage. Coverage of young children exceeded the MDG and RBM targets but not the PMI target for protection of under-fives with usage rates exceeding 80% [5-7]. While coverage of adults and older children was lower than that of the vulnerable groups that were targeted with the bulk of the subsidies, overall coverage of the entire population as a whole was more than sufficient to achieve major communal reduction of malaria transmission [9,10] if new long lasting treatments could make most nets insecticidal for their lifetime. Although only short-lived insecticide formulations were available at the time, approximately one third of the population used a recently treated net in 2006 so appreciable communal suppression is likely to have been achieved [10,31]. It is expected that such invaluable community-level benefits to be improved upon by the superior ITN technologies which are now available [31,32] and further gains in coverage as these delivery systems become better established.

**Table 1:** Net usage the previous night in Rufiji District during 2006 household survey by age group.

Usage category	Infants	Young children	Older children	Adults	Overall
N	484	732	2024	3098	6338
Proportion use (% (95% CI))					
No nets	12.8 (10.1, 16.1)	18.3 (15.7, 21.3)	45.6 (43.4, 47.7)	40.4 (38.6, 42.1)	37.4 (36.2, 38.6)
Untreated nets^a^	38.2 (34.0, 42.6)	42.0 (38.4, 45.6)	30.0 (28.0, 32.0)	31.0 (29.4, 32.7)	32.5 (31.3, 33.7)
Recently treated nets^b^	49.0 (44.5, 53.4)	39.8 (36.3, 43.4)	24.5 (22.7, 26.4)	28.6 (27.1, 30.3)	30.2 (29.0, 31.3)
Any net^c^	87.2 (83.9, 89.9)	81.8 (78.7, 84.3)	54.5 (52.3, 56.6)	59.6 (57.9, 61.4)	62.7 (61.4, 63.8)

^a ^Untreated nets are defined as nets that were not treated at all or were not treated within six months of the interview date

^b ^Recently treated nets means nets that were treated within the last six months since the day of interview

^c ^Any net means nets including both untreated and recently treated nets.

It is noteworthy that proportional contribution of various net delivery systems varied across age groups (Pearson χ^2 ^(d.f. = 20) = 844.8122; P < 0.001) and each delivery system appears to have supported its appropriate target group (Table 2). The majority of nets used by infants were obtained through the national voucher scheme, indicating this important vulnerable group is effectively targeted by this system for delivering heavily subsidized nets through commercial distributors. Nets provided at no charge to the user during the child vaccination campaign supported half of the net coverage achieved amongst young children and over a third of coverage amongst older children. The commercial market, with no subsidy other than bundled insecticide, was the biggest source of nets in the population as a whole. Nets obtained at full market price accounted for almost two thirds of use by older children and adults who must be covered if community-level suppression of transmission is to be achieved [10]. Clearly this mix of delivery mechanisms reaching different segments of the population demonstrates that all three delivery tactics are complementary rather than competitive. Rapid attainment of high net coverage for the vulnerable population was achieved through the combined contributions of the product provision campaign and voucher subsidy while broad coverage for the rest of the community resulted largely from nets purchased on the open market at full price [21]. Viewed in this integrated manner, these data provide clear evidence that commercial markets, voucher subsidies and free net distribution are not mutually exclusive choices and can complement each other effectively to make the most of limited subsidy.

**Table 2:** Sources of nets used the previous night in Rufiji District during 2006 household survey by age group.

Bed net source	Infants	Young children	Older children	Adults	Overall
*n*	*422*	*598*	*1102*	*1848*	*3970*
*Proportion use (% (95% CI))*					
Voucher	41.5 (36.9, 46.2)	10.0 (7.9, 12.7)	4.0 (3.0, 5.3)	14.9 (13.3, 16.6)	14.0 (12.9, 15.1)
Free-Vaccine	27.0 (23.0, 31.5)	50.0 (45.8, 53.8)	37.3 (34.5, 40.2)	15.8 (14.2, 17.5)	28.1 (26.7, 29.5)
Free-Other	5.9 (4.0, 8.6)	12.7 (10.3, 15.6)	19.2 (16.9, 21.6)	8.9 (7.7, 10.3)	12.0 (11.0, 13.1)
Commercial market	24.0 (20.1, 28.2)	26.3 (22.9, 29.9)	37.9 (35.1, 40.8)	60.2 (58.0, 62.4)	45.1 (43.5, 46.6)
Unknown source	1.7 (0.8, 3.4)	1.2 (0.6, 2.4)	1.6 (1.0, 2.6)	0.2 (0.1, 0.5)	0.9 (0.6, 1.2)

Free-other means all nets that a user obtained it as a gift from a relative or a friend

Unknown source means all nets that the respondent could not identify its source

**Figure 2:**
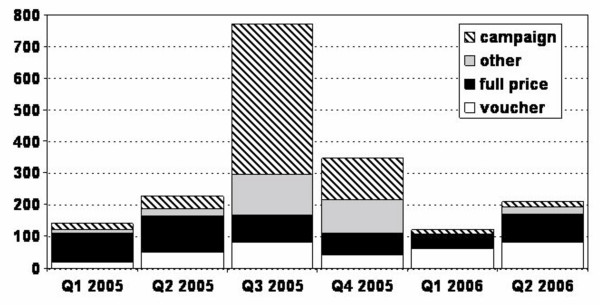
**Reported sources and time of acquisition of nets used in Rufiji District at the time of the 2006 household survey.** Note that the voucher programme was launched in late 2004 and the free distribution occurred at the start of the third quarter in 2005. The household survey did not capture complete data for the second quarter of 2006 because this is when the household surveys began.

Figure 2 shows the number of nets in use during the 2006 survey by source and time of acquisition. The distribution of nets at no cost to the recipients through the vaccination campaign in the third quarter of 2005 caused a clear surge in net acquisition. It is interesting to note that a concomitant surge was observed for nets obtained through sundry other sources, suggesting significant redistribution within families and communities. Over 16% of all nets reported for this period were obtained through this mechanism. It therefore seems likely that under-five children who already had a net and received another through free distribution from the campaign may have passed on the existing or additional net. This suggests that every additional net supplied contributes to both personal and communal protection in the population as a whole, regardless of any leakage or exchange. While the number of nets procured on the unsubsidized market or through the voucher for pregnant women declined shortly after the free distribution in mid-2005, within a year these market-based sources of nets appear to have rebounded to levels equal to or in excess of their levels before the campaign. Provision of nets at no cost through the public sector did not compromise the viability of either the voucher scheme or the commercial market, presumably because this limited full subsidy was targeted toward a previously unsubsidized population group and was not of sufficient volume to compete with established demand for nets in the entire population. Such hybrid approaches represent an excellent mix of strategies for catching up and keeping up coverage when subsidies are not adequate for mass distribution to entire populations [32,33]. This suggests that a number of options are available to NMCPs and that diverse tactics can be astutely combined to achieve the RBM, MDG and PMI targets rapidly and sustainably, even with only partial subsidies.

**Figure 3:**
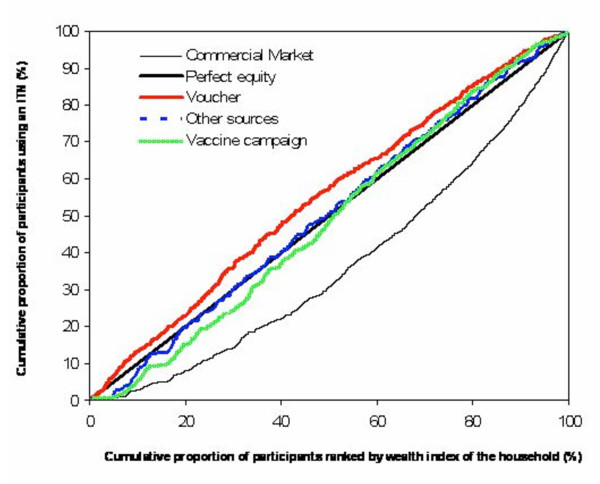
**Degree of inequality for net distribution strategies for different wealth status.** The concentration curve below the line of perfect equity indicates that net use obtained from that source is concentrated among higher socio-economic groups. The concentration curve above the line of equity indicates that net use obtained from that source is concentrated among the poor. When the curve lies along the line of perfect equity, then there is no wealth related inequity for that distribution strategy.

Table 3 demonstrates that contributions of various delivery mechanisms to net use vary by socio-economic status (Pearson χ^2 ^(d.f. = 20) = 844.8122; P < 0.001). Net coverage was far higher for the least poor with more than four fifths of this better off quintile using a net. Over half obtained their net from commercial market at full price. The concentration index of inequality was the highest for nets obtained from this almost completely unsubsidized distribution mechanism (Table 3) and the concentration curve for these nets lies below the line of perfect equity (Figure 3). This confirms favouritism of the unsubsidized market towards those who are better off and can readily pay for nets. Nevertheless, unsubsidized commercially sold nets were the most important for net coverage achieved by all other socio-economic groups except for the poorest where this source was matched but not exceeded by those obtained for free from the child vaccination campaign. Indeed, the contribution of the unsubsidized commercially-obtained nets towards net coverage achieved by the poor exceeded that of nets obtained with assistance of the voucher subsidy, demonstrating that even the poorest invested in nets for non-target groups when no subsidy was available (Table 2).

**Table 3:** Sources of nets used the previous night in Rufiji District during 2006 household survey by socioeconomic status

Bed net source	Most poor	Very poor	Poor	Less poor	Least poor	Concentration Index
N (6323 overall)^a^	985	1249	1398	1357	1334	
*Proportion use (% (95% CI))*						
No net	66.8 (63.8, 69.7)	42.5 (39.8, 45.3)	36.6 (34.1, 39.2)	30.1 (27.8, 32.6)	19.0 (17.0, 21.2)	-0.214 (-0.335, -0.093)
Voucher	5.8 (4.5, 7.4)	8.3 (6.9, 10.0)	9.4 (8.0, 11.0)	10.6 (9.1, 12.4)	8.9 (7.4, 10.5)	0.067 (-0.027, 0.161)
Free-Vaccine	11.2 (9.3, 13.3)	19.6 (17.5, 21.9)	20.7(18.7, 23.0)	20.2 (18.1, 22.4)	14.4 (12.6, 16.4)	0.015 (-0.129, 0.159)
Free-Other	6.2 (4.8, 7.9)	8.6 (7.1, 10.3)	8.1 (6.8, 9.6)	7.1 (5.9, 8.6)	7.1 (5.8, 8.6)	-0.005 (-0.074, 0.064)
Commercial market	9.8 (8.0, 11.8)	20.4 (18.3, 22.7)	24.1 (21.9, 26.4)	31.6 (29.2, 34.1)	50.3 (47.6, 53.0)	0.254 (0.119, 0.389)
Unknown source	0.3 (0.1, 0.9)	0.6 (0.3, 1.2)	1.1 (0.6, 1.8)	0.3(0.1, 0.8)	0.4 (0.2, 0.9)	-0.045 (-0.295, 0.205)
Any source^b^	33.3 (30.3, 36.1)	57.5 (54.7, 60.2)	63.4 (60.8, 65.9)	69.9 (67.3, 72.1)	81.1 (78.8, 83.0)	0.127 (0.021, 0.234)

^a ^Do not add up to 6338 (see tables 1) because data on households' assets and housing characteristics were collected in forms that were separate from those used for other variables. These two sets of forms were merged using household registration numbers that were supposed to be identical for forms that were related. Some of these related forms were erroneously filled with different numbers that could not be rectified and therefore the study participants had to be dropped for this analysis as they could not be assigned their rightful economic status.

^b ^Proportion of nets obtained from all shown net sources

Conventional interpretation of concentration indices, comparing coverage of the poorest with that of the least poor, might suggest that all forms of subsidized delivery (voucher and free product provision) are completely equitable (Table 3). Similarly, uncritical interpretation of Figure 3 might confirm this observation with all these forms of subsidized delivery approximating the line of perfect equity. Closer examination of Table 3, however, indicates that the least poor may not be a representative group with which to compare the poorest because this group appears to underutilize subsidized delivery mechanisms and relies more heavily upon the open market, presumably for reasons of choice and convenience. Comparing the poorest with the three intermediate wealth quintiles shows that the former do suffer substantial inequities relative to the latter, regardless of what system is used to deliver subsidized nets. While coverage of the three intermediate wealth quintiles is relatively even, coverage of the poorest is consistently and substantially lower for both voucher-subsidized and freely distributed nets. Nevertheless, population-wide net coverage for the most poor approaches the levels at which community level protection may be achieved [10] when the reported long lasting net treatment technologies[30] will make all the nets insecticidal. It also should be noted that the poorest are typically intermingled with neighbours from better socioeconomic strata and, therefore, share the communal protection delivered by the coverage of these groups which is typically twice as high.

## Discussion and conclusion

This study has reported how commercial markets, free product distribution through mass campaign and voucher subsidy can work together to achieve high rapid and sustainable ITN coverage in a poor rural African community exposed to intense and perennial malaria transmission. Some recent publications have advocated that ITN coverage targets can only be achieved through mass distribution at no cost to the end user [15-17]. While these arguments are based largely on opinion, this study provides empirical data showing that direct product distribution through mass campaigns can accelerate attainment of coverage in target groups if astutely integrated with market based approaches which similarly target subsides to the neediest. Vouchers and free products both clearly succeeded in targeting their respective subsidies to different biologically vulnerable population groups they were intended to support (Table 2) with insecticide treatment rates being highest in the vulnerable groups (Table 1). Not only did both approaches bias coverage of nets and insecticide treatment to the young (Table 2), in combination they enabled a largely unsubsidized private sector to flourish, resulting in high coverage of the non-vulnerable majority of the population. This latter point is crucially important for achieving community-wide suppression of transmission [9,10,12] and represents a step forward relative to recent studies in Kenya and Ghana which focused exclusively on coverage of infants and young children [32,34]. When data for infants and young children are pooled, use of any net for under-fives observed in this study is higher 1020/1216 (83.9%) than that reported from Kenya (80.3%) [34] and in Ghana (72.6%) [32]. Use of recently treated nets shown amongst under-fives appears to be lower in this study (43.4%; 528/1216) than that achieved in Kenya 67.3% and Ghana 59.6%. Nevertheless, it is suggested that the issue of net treatment rates will become less challenging as NMCPs increasingly prioritize the exclusive promotion of long-lasting insecticidal nets and treatments [12].

Importantly, largely unsubsidized nets were available to everyone able and willing to pay for them through the commercial market which was actively promoted through the voucher scheme [21]. Indeed, the majority of all nets used in our study area were obtained at full market price reflecting the contribution of the community itself to the cost of high population-wide coverage under current circumstances in which global public subsidies for malaria control amount to only 20% of the true full cost [35]. Although use of unsubsidized nets was greater amongst the least poor (Table 3), recently voiced concerns about the potential inequities associated with market-based delivery of subsidies [16,34] appear to apply just as much to free product delivery, with approximately equivalent inequity resulting from both voucher discounts and fully subsidized distribution of nets through vaccination campaigns.

Our study has shown that overall net use in Rufiji district was far higher than most other parts of Africa. This data provides further definitive evidence that net delivery strategies other than fully subsidized mass distribution to entire populations can achieve net coverage high enough to provide community level benefits. Achieving high ITN coverage for non-pregnant adults and older children is just as important as comprehensive personal protection of vulnerable groups for three reasons. First, they are the majority in the population and more attractive to mosquitoes [36-38] so reasonably high but not necessarily comprehensive net coverage is essential to deliver the mass effect of ITNs [9,10,12]. Second, they are the only source of labour for economic productivity required to support the population as a whole and dependent children in particular, so the impacts of malaria illness and associated costs trickle down to every one [39]. Third, many people living with HIV may be biologically vulnerable to severe malaria in a manner similarly to small children and pregnant women [40,41]. Here it is demonstrated that high net coverage for adults and older children has been achieved in our study area, largely through purchase of unsubsidized nets at market prices. This study supports the view that high and broad ITN coverage including older children and adults is important for effective malaria control [9-11] but show here that market-based cost-sharing strategies utilizing voucher-targeted subsidies can also help achieve this goal [16]. However, unlike previous reports from other parts of Tanzania [14], this is evidence that such targets can be achieved very rapidly by augmenting voucher-stimulated "keep-up" mechanisms with complementary "catch up" campaigns directly distributing products at no cost to the end user.

One limitation of this study is that our survey did not distinguish between pregnant and non-pregnant adult females so coverage in this key target group could not be directly assessed. Nevertheless, high net usage by infants, largely supported by the voucher scheme, presents an informative proxy for net use by pregnant women because mothers of newborns tend to share sleeping sites with their offspring in this area. All in all, Tanzania may be a uniquely informative site for a study of this kind. It was the site of initial experimental hut trials and ITN efficacy studies [19] and it is where such innovations as do-it-yourself treatment and social marketing were pioneered [42]. Moreover, the combination of both a socialist past and more recent reforms to enable a market-based economy may have elevated many of the national and local policy makers above the ideological considerations that have too often characterized discussions about how to deliver ITNs. The open market and subsidized voucher programme were relatively mature at the time the free distribution was undertaken in Rufiji District and this may not be a unique situation. Market-based delivery systems for ITNs are operational in many countries and present a valid option for attaining high coverage without comprehensive subsidies. As donor support for mass distribution of free nets become more widely available, and it is sincerely hoped that this will be viewed as potentially complementary rather than disruptive to market-based distribution and promoted as a means for rapidly expanding coverage, particularly amongst vulnerable groups. Unlike the development and implementation of the voucher programme [21], the decision to undertake a mass distribution in Rufiji District was not achieved by broad national consensus and was more opportunistic in its origins. Instead, the mix of strategies employed in Rufiji District represents the pragmatic efforts of local and national health officials to sensibly deploy scarce resources offered by partners with competing ideologies. In that sense, it is expected that Tanzania and Rufiji District will not remain an historic exception. If, decades after the life-saving value of this intervention has been firmly established [3], ITN advocates, no matter their stripe, can agree that broad community coverage is a priority, then there is promise that diverse approaches can be simultaneously applied in an imperfect but constructive and complementary manner. It is time to step away from the ideological debates about how to deliver ITNs and engage constructively with the local and national authorities to confronting this challenge through whatever means are practical.

## Authors' contributions

RAKH contributed to the design of the study, supervised the field surveys, analysed and interpreted the data, and wrote the manuscript in consultation with the other authors. GFK contributed to the analysis and interpretation of the data and to the drafting and editing of the manuscript. EK, PDM and AM contributed to the analysis and interpretation of the data and to the editing of the manuscript. RPMG assisted in the design of the study, execution of the field surveys, interpretation of the data and editing of the manuscript. SMKA and SPK oversaw all aspects of the study, including design and execution of the field work, analysis and interpretation of the data and drafting of the manuscript.
